# The Effect of Family Atmosphere on Chinese College Students’ Pro-social Behavior: The Chained Mediation Role of Gratitude and Self-Efficacy

**DOI:** 10.3389/fpsyg.2022.796927

**Published:** 2022-04-12

**Authors:** Na Li, Qiangqiang Li

**Affiliations:** School of Humanities, Tongji University, Shanghai, China

**Keywords:** family atmosphere, pro-social behavior, gratitude, self-efficacy, college students

## Abstract

The current study aimed to explore how family atmosphere influenced pro-social behavior among Chinese college students and to explore the mediation roles of gratitude and self-efficacy. We recruited 800 Chinese college students, and the participation rate was 89% (712 participants, *M* = 19.26, *SD* = 1.23). Participants completed the family atmosphere scale, the pro-social tendencies measure, the gratitude questionnaire, and the general self-efficacy scale. Results indicated that (1) Family atmosphere, gratitude, self-efficacy, and pro-social behavior were positively correlated after controlling for the grade, gender, and age. (2) The family atmosphere affected pro-social behavior not only directly, but also indirectly through the partial mediating role of gratitude and self-efficacy. Moreover, gratitude and self-efficacy also played a full chained mediation role in the relationship between the family atmosphere and pro-social behavior of college students. Therefore, a supportive family atmosphere is conducive to promoting college students’ gratitude and self-efficacy, in turn affecting their pro-social behavior.

## Introduction

Pro-social behavior refers to behaviors that are beneficial to others in our daily lives, such as charitable donations, organ donations, and volunteering behaviors (Eisenberg et al., 1995). Pro-social behavior can promote individual life satisfaction, happiness, mental health, and other psychological states (Miles et al., 2021). Understanding the mechanisms of college students’ pro-social behavior will help to improve their mental health and wellbeing. Therefore, researchers have paid much attention to the pro-social behaviors of college students and explored the factors that influence pro-social behaviors (Maiya et al., 2021; Sunbul, 2021).

The bioecological theory suggests that the psychosocial individuals’ development can be explored from a multidimensional and complex system that examines both environmental and individual factors, rather than using any single factor, i.e., emphasizing the important influence of the combined effect of environmental and individual factors on human psychological and behavioral development. The bioecological theory states that environmental factors consist of four interactive systems, namely, the macrosystem, the outer system, the mesosystem, and the microsystem (Rosa and Tudge, 2013). According to the bioecological theory, the formation and development of pro-social behavior of college students depend not only on individual factors but also on the environmental system with which they are in contact. The family, as the first major environmental system of human development, plays a very important role in the development of individuals. Especially, when individuals live in a warm and supportive family atmosphere, it is very constructive for their mental health (Mesurado and Richaud, 2017). The family atmosphere is a dimension of the family dynamics system that reflects the organization and patterns of communication and interaction within a family system (Kuisma et al., 1997). Previous researchers have found that family has a significant impact on individuals’ pro-social behavior. For instance, Mesurado and Richaud (2017) showed that the combination of parental support has an important influence on pro-social flow and positive behavior such as pro-social behavior toward friends and family. Ferreira et al. (2016) suggested a positive link between the quality of relationships with early caregivers and children’s pro-social behavior. These results indicated that individuals living in a harmonious family environment have higher altruistic tendencies and a good and pleasant family atmosphere reflects good family function. Therefore, we propose hypothesis 1: the family atmosphere of college students predicts their pro-social behavior.

In addition to family factors, individual factors can also influence pro-social behavior. The pro-social behavior theory suggests that there are two critical individual factors affecting pro-social behavior (Eisenberg et al., 1995). First, individuals need to perceive that others need help, which is an important condition for pro-social behavior to occur. Gratitude is related to perceiving that others need help, and it has been suggested that gratitude promotes individuals’ pro-social motivation (Bartlett and DeSteno, 2006; Grant and Gino, 2010; Williams and Bartlett, 2015). McCullough et al. (2002) concluded that individuals with a strong tendency to be grateful have a lower threshold for experiencing gratitude in daily life and can perceive help-seeking signals from others. You et al. (2020) examined the association between gratitude and pro-social behaviors in a sample of adolescents, the results indicated that gratitude influenced pro-social behaviors. Similarly, Shoshani et al. (2020) conducted three experiments that showed that gratitude positively affected preschool children’s pro-social behavior. Moreover, Oliveira et al. (2021) found that the gratitude intervention would increase state gratitude and, consequently, increase positive affect and empathic concern, and decrease negative affect, leading to increased intentions to engage in pro-social behaviors during the COVID-19 pandemic. In addition, a good family atmosphere is positively related to gratitude. Previous research has shown that a positive family atmosphere is conducive to the development of individual gratitude. Specifically, Lam and Chen (2021) indicated that individuals with healthy family interaction, as indicated by perceived better family communication, mutuality, and harmony with family members, tended to report higher general gratitude. Accordingly, we propose hypothesis 2: gratitude mediates the association between the family atmosphere and pro-social behavior.

Second, whether an individual will engage in pro-social behavior is also related to the individual’s sense of self-efficacy (Liu and Ngai, 2019; Sun et al., 2019). In other words, individuals are more likely to engage in pro-social behavior when they are aware of others’ help signals and believe they are capable of helping the other person. Self-efficacy is defined as a person’s belief in their ability to exercise control over their behaviors (Wood and Bandura, 1989). Barrows et al. (2013) noted that individuals with high self-efficacy are confident in their abilities that they seek opportunities to increase their knowledge and skills to succeed at a particular task and that individuals mobilize the necessary motivation, cognitive resources, and a range of actions in the process. Barrows et al.’s ideas can also be applied to pro-social behavior, as Liu and Ngai (2019) found that pro-social behavior is closely related to self-efficacy, with individuals with high self-efficacy showing more pro-social behavior. In addition, a good family atmosphere is positively related to self-efficacy. Previous research has shown that a positive family atmosphere is conducive to the development of individual self-efficacy. Specifically, Wright et al. (2020) noted that good family relationships are beneficial to individuals’ self-efficacy. Thus, we believe that a harmonious and good family atmosphere can promote college students to feel more attention and support, develop higher gratitude, acquire higher self-efficacy, and thus be more likely to engage in pro-social behaviors. Thus, we propose hypothesis 3: self-efficacy mediates the association between the family atmosphere and pro-social behavior.

Additionally, evidence suggests that as a positive psychosocial attribute, gratitude plays an essential role in self-efficacy. Datu and Yuen (2020) found that gratitude could positively predict individual self-efficacy. Studies have emerged indicating that gratitude can improve health behaviors (Cousin et al., 2020) and academic achievements (Datu and Yuen, 2020) by enhancing individuals’ self-efficacy. However, few empirical studies are exploring the link of gratitude to self-efficacy concerning pro-social behaviors. Accordingly, we believe that grateful individuals also have a high sense of self-efficacy, which in turn promotes more pro-social behavior. Taken together, we propose hypothesis 4: gratitude and self-efficacy play a chain mediating role in the relationship between the family atmosphere and pro-social behavior.

**FIGURE 1:**
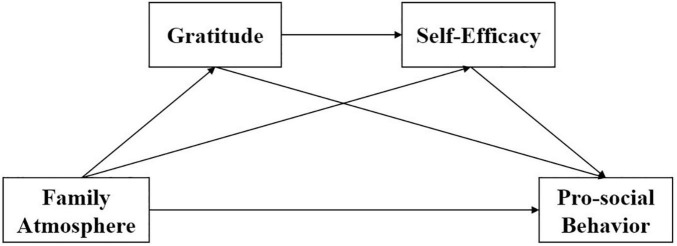
Hypothetical model of the relationship between the family atmosphere, gratitude, self-efficacy, and pro-social behavior.

In sum, pro-social behavior will help to enhance the individuals’ mental health, and family atmosphere is also crucial for the psychological development of individuals, while previous studies have rarely investigated how family atmosphere affects pro-social behavior. Thus, the current study focused on the relationship between the family atmosphere and pro-social behavior of college students as well as the chained mediating effect of gratitude and self-efficacy. Taken together, based on theory and the hypotheses of the current study, a theoretical model of the chained mediating effect of the family atmosphere and pro-social behavior was constructed, and the model was shown in Figure 1.

## Methods

### Participants and Procedure

A total of 800 college students were recruited from an eastern Chinese city as participants. In the present study, two universities were first contacted, and they were informed of the purpose of this study. This study received approval to recruit all college students based on their voluntary participation. After excluding the questionnaires with incomplete answers, the number of valid questionnaires was 712 (these students’ majors include sociology, law, chemistry, physics, and exclude psychology majors), and this included 301 subjects in the first year (171 males and 130 females), 188 subjects in the second year (102 males and 86 females), 119 subjects in the third year (79 males and 40 females), and 104 subjects in the fourth year (66 males and 38 females). The age range of all subjects was 18–22 years (*M* = 19.26 years, *SD* = 1.23, male 58.7%). Participants received a souvenir after completing the questionnaire.

The present study was approved by the Research Ethics Committee of Tongji University. Before the formal investigation was conducted, all college students were informed of research purposes. We assured that all personal information and participants’ responses would be kept confidential and would be used for research purposes only.

## Measures

### Family Atmosphere

The Family Atmosphere (FA) scale (Kang et al., 2001) was included to assess participants’ family atmosphere. The FA scale consisted of 8 items (e.g., “My family members can easily express warmth and concern for each other”). Participants responded on a Likert-type scale ranging from 1 (completely disagree) to 5 (completely agree). The scale has good reliability and construct validity in previous studies (Yu et al., 2021). In the present study, the internal reliability of the FA subscale was adequate (Cronbach’s α = 0.88) and confirmatory factor analysis (CFA) suggested that the corrected model fit the data well: *χ^2^/df* = 2.08, GFI = 0.91, IFI = 0.90, CFI = 0.92, TLI = 0.88, RMSEA = 0.07.

### Pro-social Behavior

Participants’ pro-social behavior was measured using the Pro-social Tendencies Measure (PTM, Carlo and Randall, 2002). The scale consisted of 26 items (sample item: “When people are in a bad mood, I often help them”). Participants responded on a Likert-type scale ranging from 1 (not true at all) to 5 (very true of me). The scale has been used with Chinese samples and was found to have good construct validity and satisfactory internal consistency (ranging from α = 0.56 to α = 0.83) (Kou et al., 2007). In the present study, the internal reliability of the scale was adequate (Cronbach’s α = 0.81) and confirmatory factor analysis (CFA) suggested that the corrected model fit the data well: *χ^2^/df* = 1.95, GFI = 0.90, IFI = 0.93, CFI = 0.90, TLI = 0.91, RMSEA = 0.04.

### Gratitude

Participants’ gratitude was measured using the Gratitude Scale to measure gratitude (McCullough et al., 2002; Chen et al., 2008). The scale consisted of 6 items (sample item: “There are so many things in life that I feel grateful for”), and each was scored on a 7-point scale (1 represents “strongly disagree” and 7 represents “strongly agree”). The questionnaire has good reliability and construct validity in previous studies (Zhen et al., 2021). In this study, the scale had satisfactory internal consistency (Cronbach’s α = 0.83) and confirmatory factor analysis (CFA) suggested that the corrected model fit the data well: *χ^2^/df* = 2.81, GFI = 0.90, IFI = 0.91, CFI = 0.94, TLI = 0.90, RMSEA = 0.07.

### Self-Efficacy

Participants’ self-efficacy was measured using the General Self-Efficacy Scale (GSES, Scholz et al., 2002). The scale consisted of 10 items (sample item: “If I do my best, I can always solve the problem”), and each was scored on a 4-point scale (1 represents “totally incorrect” and 4 represents “totally correct”). The scale has good reliability and construct validity in previous studies (Chu et al., 2019). In this study, the scale had satisfactory internal consistency (Cronbach’s α = 0.86) and confirmatory factor analysis (CFA) suggested that the corrected model fit the data well: *χ^2^/df* = 2.81, GFI = 0.90, IFI = 0.91, CFI = 0.94, TLI = 0.90, RMSEA = 0.07.

## Data Analysis

In this study, SPSS 22.0, PROCESS, and Amos 24.0 software were used for data analysis. We conducted a confirmatory factor analysis (CFA) of family atmosphere, gratitude, self-efficacy, and pro-social behavior questionnaire scores using Amos 24.0 software, and the results showed that the modified model matched the data well.

SPSS 22.0 was used for data analysis. We examined the Means, standard deviations, and correlations among variables. Since family atmosphere, gratitude, self-efficacy, and pro-social behavior were all measured by self-reported scales, there was a possibility that this may lead to common method bias effects. Therefore, to avoid common method bias in self-assessment analysis and to improve the authenticity of the participants’ responses, all questionnaires were completed anonymously in the sample tests. We used Harman’s single-factor test to carry out the exploratory factor analysis of 50 items of the three scales. A total of 12 factors with feature roots greater than 1 were extracted. The explanatory power of the first factor was only 24.38%, which is less than 40% of the criteria. Therefore, no common method bias was observed in the present study.

In addition, the PROCESS macro for SPSS was used for mediation model analysis (Hayes, 2013). We use model 6 in PROCESS to estimate the effects in the mediation models while controlling for participants’ age, grade, and gender. We set the gender and age of the individual as control variables because previous studies have shown that these variables are significantly associated with pro-social behavior (Eagly and Alice, 2009). Additionally, the individuals in the sample were from different grades, so to avoid additional effects of grade variables on the results (Jiang et al., 2021), we eventually kept the grade, gender, and age of the participants in a statistical model for control.

## Results

### Pre-analysis

A total of 800 questionnaires were distributed, and we excluded questionnaires with missing values and finally obtained 712 questionnaires with valid data. In SPSS, we used skewness and kurtosis for normality tests and also calculated their *Z*-scores, i.e., skewness *Z*-score = skewness value/standard error and kurtosis *Z*-score = kurtosis value/standard error. At the test level of α = 0.05, the data are considered to obey a normal distribution if the *Z*-score is between ±1.96. The results indicated that the samples were normally distributed, allowing for the next step of the analysis.

Additionally, validity and reliability analyses were conducted. First, average variance extracted (AVE) and composite reliability (CR) were used to examine convergent validity. Results indicated that the AVE values were all above 0.45 (Netemeyer et al., 2003) and the CR values were all greater than 0.70 (Nunnally, 1978), which suggests the acceptable convergent validity of the constructs. Second, discriminant validity was verified using a Fornell–Larcker test, that is, whether the square root of AVE of each construct was higher than the correlation coefficients with other constructs. Results showed that the square root of each construct AVE was greater than its correlation with other constructs. The results of our analysis showed in Table 1.

**TABLE 1:** Descriptive statistics, correlations, composite reliability, and average variance extracted of the main variables.

Variables	CR	AVE	*M*	*SD*	1	2	3	4	5	6	7
1. Age	−	−	19.26	1.23	1						
2. Grade	−	−	2.04	1.08	0.08	1					
3. Gender	−	−	0.41	0.49	0.09	0.16*	1				
4. Family atmosphere	0.76	0.66	3.64	1.76	–0.02	–0.06	–0.03	1			
5. Gratitude	0.94	0.58	5.45	1.01	–0.01	–0.01	–0.05	0.36**	1		
6. Self-efficacy	0.93	0.68	2.49	0.62	0.17*	–0.08	0.11*	0.42***	0.32**	1	
7. Pro-social behavior	0.94	0.63	3.60	0.59	0.02	–0.06	–0.01	0.24**	0.39**	0.39**	1

*N = 712; CR, composite reliability; AVE, average variance extracted; Gender was dummy coded such that 0, male; 1, female; Grade was dummy coded such that first year, 1; second year, 2; third year, 3; last year, 4.*

*p < 0.05; **p < 0.01; ***p < 0.001.

### Descriptive Statistics and Correlation Analysis

Descriptive statistics and Pearson’s correlations for the variables are presented in Table 1. We found that family atmosphere was positively correlated to gratitude, self-efficacy, and pro-social behavior; gratitude was positively correlated to self-efficacy and pro-social behavior, and self-efficacy was positively correlated to pro-social behavior. It can be seen that the more pleasant and harmonious the family atmosphere is, the higher the gratitude and the stronger the self-efficacy of college students, the more they tend to show pro-social behaviors.

### Examination of Mediating Effects

The present study used SPSS 22.0 with the SPSS macro program PROCESS developed by Hayes (2013) for data analysis. Referring to the statistical methods in previous studies (Xu et al., 2021), we use model 6 in PROCESS to test the chained mediation model hypothesis. Mediating effects were tested by estimating 95% confidence intervals for mediating effects by 5000 sample sampling, controlling for the age, grade, and gender of the participants.

The results showed (see Table 2 for details) that family atmosphere significantly and positively predicted pro-social behavior (β = 0.24, *p* < 0.01), this supports hypothesis 1; family atmosphere significantly and positively predicted gratitude (β = 0.36, *p* < 0.001); and family atmosphere significantly and positively predicted self-efficacy (β = 0.35, *p* < 0.001). When family atmosphere, gratitude, and self-efficacy entered the regression equation simultaneously, family atmosphere did not positively predict pro-social behavior (β = 0.01, *p* > 0.05), self-efficacy significantly positively predicted pro-social behavior (β = 0.29, *p* < 0.01), and gratitude was a significant positive predictor of pro-social behavior (β = 0.29, *p* < 0.01).

**TABLE 2:** Regression analysis of the main variables.

Variables	Pro-social behavior		Gratitude		Self-efficacy		Pro-social behavior	
	β	*t*	β	*t*	β	*t*	β	*t*
Gender	–0.04	–0.86	0.06	1.39	–0.02	–0.46	–0.05	–1.34
Age	0.04	–0.45	0.18	2.06	0.29	3.43**	–0.11	–1.26
Grade	–0.03	–0.29	–0.21	–2.28	–0.13	–1.50	0.08	0.97
Family atmosphere	0.24	6.59**	0.36	10.33***	0.35	9.95***	0.01	0.26
Gratitude					0.19	5.54**	0.29	8.20**
Self-efficacy							0.29	7.75**
*R* ^2^	0.06		0.14		0.25		0.23	
*F*	11.55***		28.17***		45.96***		35.87***	

**p < 0.01; ***p < 0.001.

**FIGURE 2:**
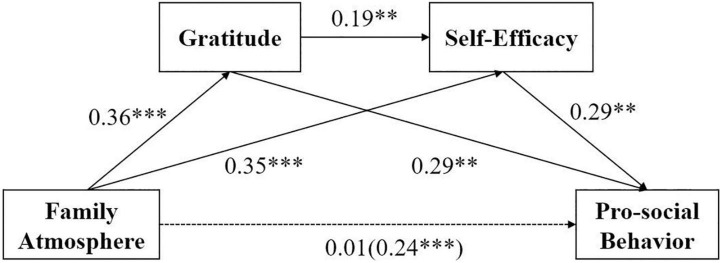
The mediating effects model after controlling for grade, gender and age.

The results of mediation effects indicated that gratitude and self-efficacy play a significant mediation role between the family atmosphere and pro-social behavior, with an overall standardized mediation effect value of 0.245. The mediation effect consists specifically of indirect effects resulting from three paths, family atmosphere gratitude pro-social behavior (effect value = 0.114), family atmosphere self-efficacy pro-social behavior (effect value = 0.110), and family atmosphere gratitude self-efficacy pro-social behavior (effect value = 0.021). The 95% confidence intervals for the above indirect effects do not contain zero, indicating that gratitude and self-efficacy were the mediating variables for the family atmosphere of college students to influence pro-social behavior, and these results support hypotheses 2–4. Moreover, The indirect effects comparison option in Model 6 was chosen to compare the indirect effects of the different paths in a two-by-two manner to examine whether there is a significant path difference: Comparison 1 shows that the Bootstrap 95% confidence interval for the difference between indirect effect 1 and indirect effect 2 does not contain a value of 0, indicating that there is a significant difference between indirect effect 1 and indirect effect 3; using the same method, there is a significant difference between indirect effect 2 and indirect effect 3 (comparison 2), and there is no significant difference between indirect effect 1 and indirect effect 2 (comparison 3) (see Table 3 and Figure 2 for details).

**TABLE 3:** Bootstrap analyses of mediating effects.

	Indirect effect	*Boot SE*	*Boot LLCI*	*Boot ULCI*	Relative mediation effect
Total indirect effect	0.245	0.031	0.187	0.308	96.08%
Indirect effects 1	0.114	0.022	0.075	0.162	46.53%
Indirect effects 2	0.110	0.017	0.074	0.148	44.90%
Indirect effects 3	0.021	0.065	0.011	0.036	8.57%
Comparison 1	0.014	0.003	0.009	0.020	
Comparison 2	0.014	0.003	0.008	0.020	
Comparison 3	0.007	0.005	–0.008	0.010	

*Boot SE, Boot LLCI, and BOOT ULCI refer to the standard error, lower and upper limits of the 95% confidence interval of the indirect effects estimated by the bias-corrected percentile Bootstrap method, respectively; Indirect effect 1: Family atmosphere Gratitude Pro-social behavior; Indirect effect 2: Family atmosphere Self-efficacy Pro-social behavior; Indirect effect 3: Family atmosphere Gratitude Self-efficacy Pro-social behavior.*

## Discussion

### The Relationship Between the Family Atmosphere and Pro-social Behavior

This study explored the relationship between the family atmosphere and pro-social behavior and its internal psychological mechanisms of action in the context of bioecological theory. The family atmosphere significantly and positively predicted pro-social behaviors among college students, which is consistent with the results of previous studies (Mesurado and Richaud, 2017). The family atmosphere affected pro-social behavior indirectly through the partial mediating role of gratitude and self-efficacy. Moreover, gratitude and self-efficacy also played a full chained mediation role in the relationship between the family atmosphere and pro-social behavior of college students. This result supports the bioecological theory that family atmosphere as a microsystem variable can not only have a direct effect on pro-social behavior but also indirectly affect pro-social behavior by influencing individual-level variables (gratitude, self-efficacy).

### Independent Mediation of Gratitude and Self-Efficacy

The family atmosphere can influence pro-social behavior indirectly through gratitude, supporting the previous findings (Amaro and Miller, 2016; du Plessis et al., 2020). The results may be explained in two ways. On the one hand, individuals in a good family interaction pattern are more inclined to show higher levels of gratitude because of the perception of better family communication and positive relationships between family members. In other words, a positive and pleasant family atmosphere is conducive to cultivating individual gratitude, and a healthy family interaction pattern can help individuals recognize the importance of gratitude and make it easier for them to experience gratitude through interactions among members (Lam and Chen, 2021), thus increasing their level of gratitude. On the other hand, most Chinese college students face the challenges of life alone without their parents during the transition from secondary school to college. College students in the transition period need support and assistance from their families and family members, and individuals who previously lived in a pleasant and positive family atmosphere may be more likely to develop gratitude experiences of mutual care among family members. The gratitude experience will also indirectly provide psychological support for college students to live independently, and may also translate the gratitude experience into interaction patterns with others, prompting individuals to experience more security and trust, and thus exhibit pro-social behaviors (Amaro and Miller, 2016).

The family atmosphere can influence pro-social behavior indirectly through self-efficacy. A positive and pleasant family atmosphere is conducive to good interaction among family members, and this positive interaction pattern will have a positive influence on individuals (Ma et al., 2007; Yu et al., 2021). This positive influence is mainly manifested in the higher level of self-efficacy that individuals have in being able to handle things well in their lives. Individuals are more susceptible to positive, optimistic interaction patterns during the process of receiving psychological support, which in turn can increase self-efficacy (Weiser and Riggio, 2010). One of the important factors in determining whether an individual shows pro-social behavior is the individual’s perceived ability to engage in pro-social behavior, and college students in a good family atmosphere are more likely to engage in pro-social behavior due to higher self-efficacy, and the results of the current study also support the results of previous empirical studies on the family atmosphere and pro-social behavior (Wrzus et al., 2011; Mesurado and Richaud, 2017).

In addition, the results showed that the gratitude and self-efficacy mediated effects were not significantly different. The path analysis revealed that the positive predictive effect of the family atmosphere on gratitude was not significantly different from the predictive effect on self-efficacy, and the positive predictive effect of gratitude on pro-social behavior was not significantly different from that of self-efficacy. The possible explanation for this result is that gratitude (perceiving others’ needs) and self-efficacy, as two important factors influencing pro-social behavior, are equally important in influencing pro-social behavior. Thus, gratitude is as closely related to pro-social behavior as it is to self-efficacy.

### The Chained Mediating Role of Gratitude and Self-Efficacy

Gratitude positively predicted self-efficacy, which is consistent with previous research findings (Datu and Yuen, 2020). The relationship between gratitude and self-efficacy can be explained in the following three ways. First, highly grateful individuals are more likely to perceive and receive more social support from others (including family and friends, and even strangers) in their lives, and can experience more care (Wood et al., 2008). Once an individual receives care and social support from others, he or she enhances his or her psychological capital, which can improve the ability to cope with frustration and enhance self-efficacy more easily. Second, gratitude, as one of the positive emotions, can broaden an individual’s perspective, enrich his or her way of thinking, and make it easier to focus on the positive aspects of things (Fredrickson and Joiner, 2002), making the individual’s cognition and behavior more adaptive, thus improving their self-efficacy. Finally, as a positive emotion, gratitude increases the accessibility of positive information in memory so that gratitude activates positive evaluations of the self. For pro-social behavior, gratitude can lead to more positive information about students’ ability to engage in pro-social behavior and, therefore, may lead to increased levels of self-efficacy, and individuals with high self-efficacy tend to have higher levels of confidence about engaging in pro-social behavior, which in turn increases the propensity to engage in pro-social behavior and is more likely to engage in actual pro-social behavior.

## Implications

This study has several important implications. The chained mediation effect in this study reveals the interaction mechanism of family atmosphere, gratitude, and self-efficacy influencing college students’ pro-social behavior, which makes up for the shortage of previous studies exploring the relationship between family factors and individual factors influencing college students’ pro-social behavior, and has practical implications for enhancing college students’ pro-social behavior. On the one hand, it can be aimed at improving the family atmosphere and establishing a positive interaction pattern among family members. On the other hand, it can be aimed at developing individual gratitude and self-efficacy to enhance the social support individuals receive, improve their confidence in coping with frustration, and promote and transmit pro-social behaviors such as being helpful, coordinating interpersonal relationships, and defending the public interest.

## Conclusion

This study enriches our understanding of the relationships between individuals’ family atmosphere and pro-social behavior among Chinese college students. Our findings showed that the relation between the family atmosphere and pro-social behavior is mediated by gratitude and self-efficacy. These results help us to understand the possible mechanisms by which family atmosphere increases pro-social behavior among college students.

## Limitations and Future Directions

Finally, this study has four limitations and future directions. First, this study examined only the correlation between the family atmosphere, gratitude, self-efficacy, and pro-social behavior, so we cannot infer a causal link. Future studies can investigate a causal relationship through a more rigorous experimental design. Second, a longitudinal study design would be more effective to obtain the developmental trend of the relationship between gratitude, self-efficacy, and pro-social behavior in the context of the macro-environmental system. Third, this study used only early theories of bioecology and did not use the full theory (Vélez-Agosto et al., 2017), which could be used in future studies to better understand the formation and development of problem behaviors. Fourth, the sample of this study only included college students, which limits the generalizability of the findings of the current work to general adult populations. Future studies could collect data from more types of individuals and verify whether the results of this study can be replicated to the general population.

## Data Availability Statement

The original contributions presented in the study are included in the article/Supplementary Material, further inquiries can be directed to the corresponding author.

## Author Contributions

Both authors participated in the data collection, data analysis, writing, and revising of the manuscript.

## Conflict of Interest

The authors declare that the research was conducted in the absence of any commercial or financial relationships that could be construed as a potential conflict of interest.

## Publisher’s Note

All claims expressed in this article are solely those of the authors and do not necessarily represent those of their affiliated organizations, or those of the publisher, the editors and the reviewers. Any product that may be evaluated in this article, or claim that may be made by its manufacturer, is not guaranteed or endorsed by the publisher.
